# Humic + Fulvic acid mitigated Cd adverse effects on plant growth, physiology and biochemical properties of garden cress

**DOI:** 10.1038/s41598-021-86991-9

**Published:** 2021-04-13

**Authors:** Ertan Yildirim, Melek Ekinci, Metin Turan, Güleray Ağar, Atilla Dursun, Raziye Kul, Zeynep Alim, Sanem Argin

**Affiliations:** 1 Department of Horticulture, Faculty of Agriculture, Atatürk University, 25240 Erzurum, Turkey; 2 Department of Genetics and Bioengineering, Yeditepe University, 34755 Istanbul, Turkey; 3 Department of Biology, Faculty of Science, Atatürk University, 25240 Erzurum, Turkey; 4 Department of Horticulture and Agronomy, Kyrgyz-Turkish Manas University, Bishkek, Kyrgyz Republic; 5 Department of Agricultural Trade and Management, Yeditepe University, 34755 Istanbul, Turkey

**Keywords:** Abiotic, Plant physiology

## Abstract

Cadmium (Cd) is a toxic and very mobile heavy metal that can be adsorbed and uptaken by plants in large quantities without any visible sign. Therefore, stabilization of Cd before uptake is crucial to the conservation of biodiversity and food safety. Owing to the high number of carboxyl and phenolic hydroxyl groups in their structure, humic substances form strong bonds with heavy metals which makes them perfect stabilizing agents. The aim of this study was to determine the effects of humic and fulvic acid (HA + FA) levels (0, 3500, 5250, and 7000 mg/L) on alleviation of Cadmium (Cd) toxicity in garden cress (*Lepidium sativum*) contaminated with Cd (CdSO_4_.8H_2_O) (0, 100, and 200 Cd mg/kg) under greenhouse conditions. Our results showed that, Cd stress had a negative effect on the growth of garden cress, decreased leaf fresh, leaf dry, root fresh and root dry weights, leaf relative water content (LRWC), and mineral content except for Cd, and increased the membrane permeability (MP) and enzyme (CAT, SOD and POD) activity. However, the HA + FA applications decreased the adverse effects of the Cd pollution. At 200 mg/kg Cd pollution, HA + FA application at a concentration of 7000 mg/L increased the leaf fresh, leaf dry, root fresh, root dry weights, stem diameter, leaf area, chlorophyll reading value (CRV), MP, and LRWC values by 262%, 137%, 550%,133%, 92%, 104%, 34%, 537%, and 32% respectively, compared to the control. Although the highest H_2_O_2_, MDA, proline and sucrose values were obtained at 200 mg/L Cd pollution, HA + FA application at a concentration of 7000 mg/L successfully alleviated the deleterious effects of Cd stress by decreasing H_2_O_2_, MDA, proline, and sucrose values by 66%, 68%, 70%, and 56%, respectively at 200 mg/kg Cd pollution level. HA + FA application at a concentration of 7000 mg/L successfully mitigated the negative impacts of Cd pollution by enhanced N, P, K, Ca, Mg, Fe, Mn, Cu, Mn, Zn, and B by 75%, 23%, 84%, 87%, 40%, 85%, 143%, 1%, 65%, and 115%, respectively. In addition, HA + FA application at a concentration of 7000 mg/L successfully reduced Cd uptake by 95% and Cl uptake by 80%. Considering the plant growth parameters, the best results were determined when HA + FA concentration was 7000 mg/L. We have shown that, it is critical to apply a humic substance with high percentage of FA, which was 10% in this study, to mitigate the adverse effects of heavy metal stress on plant growth. In conclusion, the application of HA + FA may be suggested as an effective solution for reducing the Cd uptake of the plants by stabilizing Cd in soil and preventing translocation of Cd from the roots of plant to its shoot and leaves.

## Introduction

Soil and water are valuable natural resources crucial for the sustainability of agriculture. However, anthropogenic activities severely damaged and contaminated both soil and water. One of the major problems is the discharge of sewage water and industrial wastes to agricultural areas which creates various threats by polluting the environment with heavy metals, salts, and nitrates^1^. Pollution caused by heavy metals is a serious problem since contamination of the food chain by metal pollutants pose a danger to plant, animal and human health due to the high toxicity^2^.

Plants have a natural tendency to uptake various metals through their roots. Some of them are basic plant micronutrients such as Cu^2+^, Co^2+^, Fe^2+^, Mo^2+^, Mn^2+^, and Zn^2+^, while several such as Hg^2+^, Cd^2+^, Ni^2+^, and Pb^2+^ can be detrimental to plants. Impacts of metals on plants vary from genotype to genotype^3^ and the toxicity depends on ion type, ion concentration, plant species, and plant growth stage^4,5^.

Among the pollutants, commonly referred to as "heavy metal", cadmium (Cd) is a major problem due to its mobility in the plant-soil system. Although Cd is naturally present in the environment in a small amount (0.04–0.32 mM), agricultural applications of pesticides, phosphate fertilizers, fallout and biosolids from non-agricultural industry have enriched the soil with this element^6^. Cd is very mobile and is easily adsorbed by plants^7^. Cd is particularly dangerous since plants grown in contaminated soils may adsorb Cd in large quantities without any visible signs^3^ and enter the human body by feeding^8^. Therefore, low Cd intake by plants and the physiological responses of plants to Cd pollution are crucial to the conservation of biodiversity and food safety.

Humic substances are important organic materials that directly and indirectly affect plant growth, improve the physical and chemical characteristics of soil and increase vegetative production. The solubility and bioavailability of these substances change, once they form compounds with metals. The strong bonds formed between humic substances and toxic heavy metal ions play an important role in plant production in terms of mitigating the negative effects of heavy metal stress on plant development^9^.

Leafy vegetables are high metal ion accumulators when compared with other vegetable species^10^. *Lepidium sativum*, (a.k.a. garden cress), is a leafy vegetable that grows mainly in temperate regions. Since ancient times, seeds, leaves, roots, and flowers of garden cress have been used to treat various diseases or ailments^11^. Earlier reports have pointed out that cress is sensitive to cadmium (Cd) stress. However, to our best knowledge there is no study investigating the effect of HA + FA applications on the cress grown under Cd stress. Thus, in this work, CdSO_4_.8H_2_O polluted soil (0, 100, and 200 mg Cd/kg) was treated with four doses of HA + FA (0, 10, 15, and 20 ml/L) to elucidate the effects of HA + FA on alleviation of Cd stress, enhancement of plant growth and physiological-biochemical properties of garden cress.

## Materials and methods

### Setting up the experiment

Garden cress (*Lepidium sativum* cv Helen) was grown in polyethylene pots in the greenhouse of Atatürk University, Erzurum. The greenhouse temperature was maintained at an average temperature of 22 (± 2)°C during the day, and 17 (± 2)°C at night.

The soil used in this study was sampled to a depth of 15 cm from agricultural fields in Erzurum province, Turkey (39°55′N, 41º61′E). It was dried indoors until it could be crumbled to pass through a 4 mm-sieve and a 2 mm-sieve, for pot experiments and for analyses of physicochemical properties, respectively. The soil is classified as Ustorthents according to the soil taxonomy (Soil Survey Staff 1999). The composition of soil was 33% sand, 34% silt, 33% clay, 1.2% organic matter. Other physical and chemical properties were: pH: 5.61, cation exchange capacity: 15.5 cmol( +)/kg, electrical conductivity: 1.15 dS/m, total N: 1.15%, P: 15.5 mg/kg, Zn: 2.30 mg/kg, Cu: 1.1 mg/kg, Fe: 0.5 mg/kg, Mn: 1.8 mg/kg, Cd: 0.05 mg/kg Cd, K: 2.2 cmol( +)/kg, Ca: 11.0 cmol( +) /kg, Mg: 2.25 cmol( +) /kg.

For heavy metal stress treatments, cadmium (CdSO_4_.8H_2_O) was mixed with the medium at three different concentrations (0, 100, and 200 mg/kg) and incubated for 3 weeks.

HA + FA was supplied from Humintech GmbH (Grevenbroich / Germany) as a commercial product, namely Powhumus. The content (% dry wt) of Powhumus was as follows: HA + FA: 80–85% (Humic acid 90%, Fulvic acid 10%), Potassium as K_2_O: 10–12%; total organic Nitrogen: 1.0%. Particle size of insoluble constituents was < 100 µm and pH was 9–10. The element content of Powhumus (%, mass) was C—32, H—2.8, N—1.3. and the ash content was 22.9%. The element content on ash-free basis (% wt) was C—47.1, H—3.63, N—1.69, O—47.58.

To prepare HA + FA (Powhumus) solutions 350 g Powhumus was dissolved in 1 L water (350 000 mg/L) and then diluted to obtain three different concentrations (3500, 5250, and 7000 mg/L). HA + FA solutions were applied to the soil three times a week, starting the day before planting. As a control, 0 mg/L HA + FA was used, instead 150:100:150 kg/ha NPK was mixed into the medium as basal fertilizer.

Seeds of garden cress were sown in pots filled with 1 L of three-week-incubated garden soil: sand (1:1, v:v) mixture in a 1–1.5 cm depth, with 10 seeds per pot. After the seedling emergence, four plants were left in each pot with the same appearance.

### Harvest and growth parameters

The pot study was terminated on the 50th day from seed sowing. At harvest, four plants from each sample were taken to measure stem diameter, plant height, leaf number, aerial fresh-dry weight, and root fresh-dry weight. For dry weight measurements, the plant material was kept at 70 °C for 48 h. To determine the content of proline, sucrose, MDA, H_2_O_2_, and antioxidant enzyme activity, roughly 20 g of fresh leaves were frozen in liquid nitrogen and then stored at − 80 °C. Analyses were performed in quadruplicate.

### Chlorophyll reading value (CRV as SPAD)

The chlorophyll content of the plant leaves was determined by a chlorophyll meter (SPAD—502, Konica Minolta Sensing, Inc., Japan).

### Leaf area

The leaf areas of the plants in each application were determined using a leaf area meter (CID-202 Portable Laser Leaf Area Meter, CID Bio-Science, Inc., WA, USA).

### Membrane permeability (MP)

MP was determined according to Yildirim et al.^12^.

### Leaf relative water content (LRWC)

LRWC was determined according to Yildirim et al.^12^.

### H_2_O_2_ and MDA analysis

H_2_O_2_ was determined according to Velikova et al.^13^. The content of H_2_O_2_ was calculated by using a standard calibration curve previously made by using different concentrations of H_2_O_2_. Thiobarbituric acid-reactive substances were measured as MDA, a degraded product of the lipid, which determines the lipid peroxidation. The concentration of MDA was determined from the absorbance curve, by using an extinction coefficient of 155 mmol L^−1^ cm^−1^.

### Sucrose and proline analysis

Sucrose concentration was measured by a method given by Chopra et al.^14^. Proline concentration was assayed spectrophotometrically at 520 nm^15^.

### Catalase (CAT), peroxidase (POD) and superoxide dismutase (SOD) activity

CAT, POD, and SOD activities were determined based on the method given by Sahin et al.^16^.

### Mineral analysis

Garden cress leaves were ground after being dried at 68 °C for 48 h in an oven. Determination of the total N was achieved by the Kjeldahl method using a Vapodest 10 Rapid Kjeldahl Distillation Unit (Gerhardt, Konigswinter, Germany). An inductively coupled plasma spectrophotometer (Optima 2100 DV, ICP/OES; Perkin-Elmer, Shelton, CT) was used to determine tissue P, K, Ca, Mg, Fe, Cu, Mn, Zn, B, Cl and Cd^17,18^.

### Statistical analysis

In the experiment, a randomized plot design was used and the obtained data were analyzed using SPSS 20 statistical package program. Data were subjected to variance analysis (ANOVA) and differences of means were determined by Duncan multiple comparison test.

## Results

As can be seen in Table 1, humic + fulvic acid application affected the leaf fresh, leaf dry, root fresh and root dry weights of garden cress significantly under Cd stress condition. Our results showed that Cd pollution negatively affected all parameters investigated and the negative effect increased with the increased pollution doses. Without Cd pollution, the highest leaf fresh and leaf dry weight of the plant were obtained when HA + FA was applied at a concentration of 7000 mg/L, whereas the highest root fresh and root dry weight were found when the concentration of HA + FA applied was 3500 mg/L. These findings indicate that, without Cd pollution, leaf fresh weight and dry weight increased 35% and 30%, respectively with application of 7000 mg/L HA + FA, whereas root fresh weight and dry weight increased 33% and 15% when 3500 mg/L HA + FA was applied, compared to the control (HA + FA = 0 mg/L).

**Table 1:** Effect of cadmium and HA applications on leaf fresh, leaf dry, root fresh and root dry weight of garden cress.

Cd (mg/kg)	HA + FA (mg/L)	Leaf fresh weight (g/plant)	Leaf dry weight (g/plant)	Root fresh weight (g/plant)	Root dry weight (g/plant)
0	0	3.66 ± 0.09b	0.50 ± 0.050d	2.63 ± 0.10d	0.32 ± 0.012b
	3500	4.70 ± 0.18a	0.61 ± 0.023b	3.40 ± 0.07b	0.38 ± 0.025a
	5250	4.91 ± 0.14a	0.55 ± 0.005c	3.51 ± 0.09a	0.37 ± 0.011a
	7000	4.97 ± 0.16a	0.65 ± 0.017a	3.01 ± 0.12c	0.31 ± 0.018b
100	0	2.28 ± 0.04c	0.26 ± 0.014f	1.32 ± 0.01g	0.12 ± 0.004d
	3500	2.54 ± 0.01c	0.30 ± 0.019e	1.49 ± 0.03f	0.14 ± 0.010c
	5250	2.63 ± 0.03c	0.30 ± 0.004e	1.63 ± 0.05e	0.13 ± 0.006c
	7000	2.26 ± 1.11c	0.32 ± 0.005e	1.71 ± 0.04e	0.14 ± 0.008c
200	0	0.43 ± 0.03e	0.08 ± 0.004ı	0.14 ± 0.01j	0.03 ± 0.002g
	3500	1.25 ± 0.05d	0.13 ± 0.009h	0.51 ± 0.03ı	0.05 ± 0.004f
	5250	1.11 ± 0.07d	0.14 ± 0.006h	0.50 ± 0.03ı	0.04 ± 0.001fg
	7000	1.56 ± 0.05d	0.19 ± 0.011g	0.91 ± 0.04h	0.07 ± 0.004e

The difference between the means indicated by different letters in the same column is statistically significant (Duncan multiple comparison test, P < 0.05).

With Cd pollution, leaf fresh, leaf dry, root fresh and root dry weights were affected negatively. However, the HA + FA applications alleviated the deleterious effects of the Cd to the plant. At 200 mg/kg Cd pollution, HA + FA application at a concentration of 7000 mg/L increased the leaf fresh, leaf dry, root fresh and root dry weights by 262%, 137%, 550% and 133%, respectively, compared to the control (Table 1).

The effect of HA + FA applications on the stem diameter, leaf area and CRV of the garden cress under Cd stress is presented in Table 2. Cd pollution significantly decreased the stem diameter, leaf area and CRV in the cress. The most negative effect occurred in 200 mg/kg Cd pollution. However, HA + FA applications reduced the negative effects of 100 and 200 mg/kg Cd stress on the cress. At 200 mg/kg Cd pollution, HA + FA application at a concentration of 7000 mg/L increased the stem diameter, leaf area and CRV by 92%, 104%, and 34%, respectively, compared to the control (Table 2).

**Table 2:** Effect of cadmium and HA applications on stem diameter, leaf area, CRV, MP and LRWC of garden cress.

Cd (mg/kg)	HA + FA (mg/L)	Stem diameter (mm)	Leaf area (cm^2^)	CRV (SPAD)	MP (%)	LWRC (%)
0	0	3.24 ± 0.06b	213.30 ± 4.00c	41.00 ± 1.00a	5.93 ± 0.2e	74.39 ± 1.2a
	3500	3.27 ± 0.05b	235.93 ± 4.53b	40.00 ± 1.73a	5.93 ± 0.3e	68.35 ± 1.4cd
	5250	3.21 ± 0.07b	235.21 ± 6.22b	39.67 ± 1.15ab	5.80 ± 0.2e	70.08 ± 1.8bc
	7000	3.55 ± 0.04a	236.49 ± 7.53b	40.67 ± 0.58a	5.66 ± 0.1e	69.33 ± 1.6c
100	0	2.36 ± 0.07e	139.18 ± 3.59e	35.00 ± 1.00d	34.06 ± c0.2	54.16 ± 1.5g
	3500	2.68 ± 0.04c	204.78 ± 3.65d	37.67 ± 0.57c	24.96 ± 0.3d	59.06 ± 1.7f
	5250	2.57 ± 0.05d	214.37 ± 4.92c	40.33 ± 0.58a	24.78 ± 0.4d	64.89 ± 1.5de
	7000	2.73 ± 0.04c	253.22 ± 4.99a	38.00 ± 1.01bc	23.85 ± 0.5d	73.87 ± 1.6ab
200	0	0.85 ± 0.01ı	69.75 ± 4.75g	25.00 ± 1.10f	41.94 ± 0.8a	51.92 ± 1.8g
	3500	1.18 ± 0.03h	100.48 ± 2.39f	31.00 ± 1.05e	35.06 ± 0.4bc	63.64 ± 1.9e
	5250	1.30 ± 0.04g	100.65 ± 5.72f	29.67 ± 1.15e	34.91 ± 0.2bc	58.93 ± 1.3f
	7000	1.64 ± 0.03f	142.40 ± 2.48e	34.33 ± 0.50d	37.88 ± 0.3b	68.83 ± 1.5cd

The difference between the means indicated by different letters in the same column is statistically significant (Duncan multiple comparison test, P < 0.05).

The present study showed that Cd pollution increased the MP value in the cress while decreasing the LRWC value. The highest MP and the lowest LRWC values were determined in plants treated with 200 mg/kg Cd. The HA + FA applications mitigated the adverse effects of Cd and at 200 mg/kg Cd pollution, HA + FA application at a concentration of 7000 mg/L increased the MP and LRWC values by 537% and 32%, respectively, compared to the control (Table 2).

CAT, POD, and SOD activities were found to increase with increasing Cd pollution. At 200 mg/kg Cd pollution, 7000 mg/L HA + FA application decreased CAT and SOD activities by 43% and 21% respectively, and increased POD activity by 186%, compared to the samples without HA + FA application (Table 3). Although the highest H_2_O_2_, MDA, proline and sucrose values were obtained at 200 mg/kg Cd pollution, HA + FA application at a concentration of 7000 mg/L successfully alleviated the deleterious effects of Cd stress by decreasing H_2_O_2_, MDA, proline and sucrose values by 66%, 68%, 70% and 56%, respectively at 200 mg/kg Cd pollution level (Table 3).

**Table 3:** Effect of cadmium and HA applications on CAT, POD, SOD, H_2_O_2_, MDA, proline and sucrose activities of garden cress.

Cd (mg/kg)	HA + FA (mg/L)	CAT (EU gr/leaf)	POD (EU gr/leaf)	SOD (EU gr/leaf)	
0	0	75.15 ± 3.00bc	0.53 ± 0.01g	81.72 ± 2.27gh	
	3500	72.38 ± 2.78bcd	0.52 ± 0.02g	86.86 ± 3.80g	
	5250	76.97 ± 4.04b	0.56 ± 0.03g	85.19 ± 3.35g	
	7000	75.90 ± 1.27bc	0.54 ± 0.02g	79.57 ± 1.36ı	
100	0	68.05 ± 1.22de	0.58 ± 0.01g	131.76 ± 2.42d	
	3500	71.39 ± 1.64cd	1.16 ± 0.04e	128.58 ± 2.48de	
	5250	73.69 ± 1.36bc	1.61 ± 0.09d	122.33 ± 2.85f	
	7000	56.92 ± 2.32f	1.76 ± 0.06c	126.07 ± 1.81ef	
200	0	91.29 ± 1.97a	0.78 ± 0.05f	168.57 ± 3.65a	
	3500	77.28 ± 6.19b	1.84 ± 0.03c	156.28 ± 4.55b	
	5250	65.25 ± 1.05e	2.01 ± 0.05b	137.59 ± 1.99c	
	7000	51.58 ± 3.22g	2.23 ± 0.13a	132.75 ± 2.68cd	

Cd (mg/kg)	HA + FA (mg/L)	H_2_O_2_ (mmol/kg)	MDA (nmol/g)	Proline (µg/g)	Sucrose (%)
0	0	140.67 ± 2.08f	4.34 ± 0.19de	55.33 ± 2.84fg	41.42 ± 1.99cd
	3500	135.00 ± 10.00f	4.57 ± 0.16cde	55.39 ± 1.98fg	48.49 ± 2.02b
	5250	138.88 ± 3.40f	4.65 ± 0.09cde	55.90 ± 4.39fg	39.40 ± 2.46de
	7000	136.33 ± 4.04f	3.16 ± 0.18f	52.00 ± 1.08g	28.65 ± 2.90g
100	0	467.78 ± 18.93b	9.82 ± 1.26b	126.07 ± 2.07b	44.46 ± 1.38bc
	3500	215.03 ± 7.58e	5.28 ± 0.26c	96.94 ± 0.51c	33.89 ± 2.93f
	5250	143.90 ± 3.53f	3.92 ± 0.24ef	79.55 ± 2.8d	45.14 ± 2.09bc
	7000	116.72 ± 2.53g	3.48 ± 0.15f	31.04 ± 1.38h	22.87 ± 3.15h
200	0	662.68 ± 8.93a	13.67 ± 0.92a	200.37 ± 7.13a	62.95 ± 0.32a
	3500	311.67 ± 9.24c	5.34 ± 0.13c	82.39 ± 4.12d	36.77 ± 0.68ef
	5250	246.25 ± 2.67d	5.11 ± 0.09cd	72.89 ± 3.48e	25.92 ± 1.08gh
	7000	218.94 ± 2.82e	4.37 ± 0.06de	59.33 ± 2.72f	27.60 ± 4.99g

The difference between the means indicated by different letters in the same column is statistically significant (Duncan multiple comparison test, P < 0.05).

Cd pollution decreased all mineral elements in garden cress investigated within this work, but for the cadmium itself. On the other hand, application of HA + FA, enhanced the mineral element content of the garden cress except for Cd (Tables 4, 5). The highest N, P, K, Ca, Mg, Fe, Mn, Cu, Mn, Zn, B, and Cl were obtained from 7000 mg/L HA + FA application without Cd pollution, while the lowest values were determined from 0 ml HA + FA with 200 mg/kg Cd pollution treatment. HA + FA application at a concentration of 7000 mg/L successfully alleviated the deleterious effects of Cd stress by increased N, P, K, Ca, Mg, Fe, Mn, Cu, Mn, Zn, and B by 75%, 23%, 84%, 87%, 40%, 85%, 143%, 1%, 65%, and 115%, respectively. On the other hand, HA + FA application at a concentration of 7000 mg/L successfully reduced Cd and Cl uptake, by 95% and 80%, respectively (Tables 4, 5).

**Table 4:** Effect of cadmium and HA applications on macro element content of garden cress.

Cd (mg/kg)	HA + FA (mg/L)	N (%)	P (mg/kg)	K (mg/kg)	Ca (mg/kg)	Mg (mg/kg
0	0	1.93 ± 0.04e	1931 ± 41.13d	7164 ± 108.70ef	5076 ± 184.53de	3724 ± 85.13d
	3500	2.06 ± 0.02d	2055 ± 40.08c	7596 ± 73.63e	5536 ± 58.02c	3973 ± 60.75c
	5250	2.26 ± 0.03c	2289 ± 42.92b	10,374 ± 415.07b	5276 ± 115.41d	4120 ± 55.3b
	7000	2.56 ± 0.05a	2446 ± 37.93a	13,046 ± 85.40a	6980 ± 181.85a	4570 ± 47.80a
100	0	1.21 ± 0.03h	1405 ± 40.61g	5010 ± 168.27h	4393 ± 124.57f	2560 ± 53.70h
	3500	1.33 ± 0.01g	1711 ± 17.61e	7419 ± 127.60e	4885 ± 119.71e	2655 ± 67.54g
	5250	2.22 ± 0.08c	1561 ± 49.34f	8132 ± 111.87d	5694 ± 175.13c	2970 ± 75.21f
	7000	2.36 ± 0.07b	1521 ± 19.70f	9009 ± 190.06c	6189 ± 289.64b	3100 ± 47.66e
200	0	1.17 ± 0.01h	1064 ± 58.88j	3912 ± 63.99ı	2709 ± 34.20h	1920 ± 57.81k
	3500	1.76 ± 0.03f	1265 ± 27.38ı	6217 ± 371.25g	3654 ± 160.33g	2320 ± 49.45j
	5250	1.92 ± 0.05e	1343 ± 37.88gh	6835 ± 606.48f	3603 ± 22.65g	2580 ± 58.66i
	7000	2.05 ± 0.08d	1313 ± 17.67hı	7209 ± 92.88ef	5068 ± 163.03de	2690 ± 39.40h

The difference between the means indicated by different letters in the same column is statistically significant (Duncan multiple comparison test, P < 0.05).

**Table 5:** Effect of cadmium and HA applications on micro element content of garden cress.

Cd (mg/kg)	HA + FA (mg/L)	Fe (mg/kg)	Cu (mg/kg)	Mn (mg/kg)	Zn (mg/kg)
0	0	97.75 ± 5.42d	9.93 ± 0.31d	21.44 ± 1.05b	9.33 ± 0.35c
	3500	101.21 ± 4.90cd	11.61 ± 0.64c	21.80 ± 0.73b	10.18 ± 0.57bc
	5250	148.77 ± 5.60b	15.26 ± 0.50b	21.38 ± 0.48b	10.29 ± 0.47bc
	7000	174.35 ± 8.72a	25.50 ± 0.69a	20.47 ± 2.30bc	10.83 ± 0.52b
100	0	59.20 ± 0.94h	5.82 ± 0.38f	18.19 ± 1.65de	5.86 ± 0.38e
	3500	85.82 ± 5.57f	8.33 ± 0.22e	19.67 ± 0.48bcd	6.61 ± 0.33de
	5250	100.13 ± 4.26cd	10.27 ± 0.70cd	24.41 ± 1.16a	9.94 ± 1.74bc
	7000	107.80 ± 4.85c	14.11 ± 0.77b	16.91 ± 0.93ef	12.05 ± 0.62a
200	0	50.87 ± 1.22ı	3.24 ± 0.14g	16.90 ± 0.23ef	6.48 ± 0.39de
	3500	73.88 ± 1.76g	5.47 ± 0.44f	18.80 ± 1.58cde	7.68 ± 0.47d
	5250	86.81 ± 2.49ef	5.09 ± 0.10f	15.88 ± 0.99f	9.92 ± 0.66bc
	7000	94.22 ± 2.77de	7.90 ± 0.01e	16.93 ± 0.14ef	10.72 ± 0.62b

Cd (mg/kg)	HA + FA (mg/L)	B (mg/kg)	Cd (mg/kg)	Cl (mg/kg)	
0	0	7.31 ± 0.57e	0.20 ± 0.02g	0.32 ± 0.02ef	
	3500	8.04 ± 0.46de	0.25 ± 0.01g	0.33 ± 0.01ef	
	5250	10.16 ± 0.61b	0.32 ± 0.02g	0.31 ± 0.01ef	
	7000	11.99 ± 0.70a	0.34 ± 0.03g	0.28 ± 0.02f	
100	0	4.76 ± 0.21g	43.01 ± 0.20c	0.51 ± 0.05b	
	3500	6.36 ± 0.55f	25.99 ± 0.19d	0.40 ± 0.02cd	
	5250	8.77 ± 0.37cd	7.92 ± 1.34e	0.46 ± 0.01bc	
	7000	10.01 ± 0.58b	1.12 ± 0.81d	0.43 ± 0.05cd	
200	0	4.12 ± 0.20g	96.61 ± 0.26a	1.02 ± 0.08a	
	3500	7.36 ± 0.15e	47.05 ± 0.98b	0.36 ± 0.04de	
	5250	7.47 ± 0.29e	13.98 ± 1.44e	0.20 ± 0.02g	
	7000	8.87 ± 0.10c	3.00 ± 1.13f	0.20 ± 0.04g	

The difference between the means indicated by different letters in the same column is statistically significant (Duncan multiple comparison test, P < 0.05).

## Discussion

Heavy metals often cause toxic effects on plants such as chlorosis, inhibition of growth and photosynthesis, water balance variations and nutrient assimilation, which ultimately lead to plant death^19^. Heavy metals exhibit toxicity through four suggested mechanisms in plants. These are: (i) competition with nutrient cations for adsorption at the root surface (e.g. competition of As and Cd with P and Zn, respectively, for adsorption); (ii) inactivation of sulfhydryl groups (- SH) by direct interaction, which disrupts plant’s structure and function; (iii) collapsing the function of enzymes by displacing essential cations from specific binding sites; and (iv) the production of active oxygen species (ROS), which ultimately damage macromolecules^20^.

Cd is considered an important pollutant because of its high toxicity and high water solubility. Cadmium can alter mineral uptake through its effects on the presence of minerals in the soil or through a reduction in the population of soil microbes. Cadmium may have a negative effect on stomatal conductivity, perspiration, and photosynthesis efficiency. Chlorosis, leaf folds, and scrub are the common and easy-to-see symptoms of cadmium toxicity of plants. Cd also decrease nitrate uptake and its transport from the roots to the shoots by inhibiting nitrate reductase activity^21^.

In this study, Cd stress was found to affect the growth of garden cress negatively (Table 1). While the LRWC decreased in the cress grown under Cd stress, the MP increased (Table 2). Similarly, previous studies showed the negative effects of Cd stress on plant growth characteristics in radish^22^, garden cress^23,24^ and lettuce^25^.

On the other hand, HA + FA applications in cress grown under Cd stress positively affected the plant growth (Table 1). Furthermore, HA + FA increased LRWC and decreased MP (Table 2). Similarly, it was reported in previous studies that HA + FA applications mitigated the negative impact of heavy metal stress on plant growth properties in lettuce^26^ (Haghighi et al. 2010), radish^22^, curly lettuce^9^, triticale^27^, corn^28^ and wheat^29^. Humic substances are natural organic polyelectrolytes found in humus, which stabilize organic matter in the soil. Many authors have reported the ability of humic substances to increase the growth of different plant species that grow under different stress conditions^27^. HA + FA positively affect plant growth and yield, directly or indirectly, by improving some physical and chemical properties of the soil. Moreover, HA + FA changes the solubility and bioavailability of toxic heavy metals by forming compounds with them through strong bonds which in turn reduce the heavy metal stress on plant growth^9^. These strong bonds formed with heavy metals are a result of cation exchange capacities (CEC) of humic and fulvic acids. Owing to the high number of carboxyl and phenolic hydroxyl groups in their structure, humic substances have high cation exchange capacities (CEC), which are 600–890 cmol( +)/kg and 1000–1230 cmol( +)/kg for humic acid and fulvic acid, respectively^30,31^. These capacities are 5 to 100 times higher than that of common clay minerals, which makes humic substances perfect stabilizing agents. In addition, although there are various methods and techniques that have been used for stabilization of Cd, Pb, Cu, and Zn, application of humic substances is the only non-chemical and natural method.

In this work, in the absence of Cd stress, no change in the CAT activity was observed due to the application of HA + FA. On the other hand, the effect of Cd stress on CAT activity was found to vary with the concentration of Cd applied for control samples (HA + FA = 0), i.e. decreased when the Cd pollution level was 100 mg/kg, however increased once the Cd concentration was changed to 200 mg/kg. Although CAT activity reached a very high level in 200 mg/kg Cd polluted control sample (around 90 EU/gr leaf), application of HA + FA at a concentration of 7000 mg/L decreased CAT activity significantly, i.e. to the levels (around 50 EU/gr leaf) even lower than when no Cd stress was present (around 75 EU/gr leaf) (Table 3).

Our results also showed that, Cd stress conditions increased POD and SOD activity in garden cress. POD activity was increased even more with HA + FA applications whereas and SOD activity was decreased with applications of HA + FA (Table 3).

It has been shown in different studies that various environmental stresses (salinity, water deficit stress and heavy metal stress) affect the enzyme activity^32^. Shao et al.^33^ showed that antioxidant activity enhanced under stress conditions. On the other hand, Sergiev et al.^27^ suggested that heavy metal applications did not have a significant effect on CAT activity, whereas it increased SOD, GST (glutathione-S-transferase), and GPOX (guaiacol peroxidase) activity in triticale. Similar to our results, Ozkay et al.^9^ indicated that heavy metal stress increased SOD activity in curly lettuce, and HA applications supported this increase. The researchers reported that HA applications led to a further increase in SOD and GST activity, which served as a scavenger of reactive oxygen species. This positive effect of HA may most likely be due to the possibility of forming chelating complexes with HA in the nutrient medium or in the plant. Studies showed that humic acid and fulvic acid efficiently immobilize heavy metals and to a larger extent in the mineral soil^34^. Retention capacity was directly related to the amount of added fulvic acid content in the humic substance. The mobility of fulvic acid (FA) is higher due to its smaller size (molecular weight) and higher oxygen content which is twice of HA. In addition, FA is much more reactive because of the presence of many carboxyl (COOH) and hydroxyl (COH) groups (ranges from 520 to 1120 cmol (H +)/kg) in its structure, which makes the exchange capacity of FA more than double that of HA.

The behavior of metals in the soil was affected by humic and fulvic acid application. The effects of HA and FA on Pb, Cd, Ni in soil and availability to plants have been extensively investigated. But, inconsistent findings have been reported due to the complex nature of HA and FA and substantial differences in soil characteristics^35–37^.

Earlier reports have indicated that humic and fulvic acid treatments affected plants by affecting the exchangeable nutrient forms in the soil^35^ and decreased Pb, and Cd accumulation^38^. They suggested, humic and fulvic acid can be employed to immobilize Pb and Cd in soil. Studies conducted generally focused on the effects of humic acid, while leaving the effects of fulvic acid in humic-made content unevaluated. Previous studies reported the inhibitory effects of humic substances on heavy metal in acidic soils^39–43^. On the contrary, in alkaline soils, humic substances were found to stimulate the metal availability^36,37^. This contradiction can be explained by the unnoticed effect of fulvic acid in the humic content. The underlying reason why the prohibitive impacts of humic substances on metal availability were found at low pH is that the bond formation between metals and fulvic acid dominated under acidic conditions. In fact, in cases where the fulvic acid content in humic compounds is 5% or more, the capacity of heavy metal binding and fixing is particularly high in neutral and light acid soils. On the contrary, humic bits of humic acid content, which is very high compared to fulvic acid content, increases the availability of heavy metal. Thus, it can be said that humic acids are effective for heavy metal bioremediation because humic acids can interrelate with metals to form metal–humic complexes^34^. On the other hand, fulvic acids have been reported to inhibit metal availability and might be employed to decrease metal accumulation in the polluted acidic soils. Humic acid had a stimulating impact on heavy metal presence and were very good for metal bioremediation in alkaline soils^36,37^.

As a result, Cd pollution negatively affected plant growth parameters such as plant fresh and dry weight. However, HA + FA applications have been found to reduce this negative effect Considering the plant growth parameters, the best results were determined when HA + FA concentration was 7000 mg/L. We have shown that, it is critical to apply a humic substance with high percentage of FA, which was 10% in this study, to mitigate the adverse effects of heavy metal stress on plant growth.
