# Author Correction: Humic + Fulvic acid mitigated Cd adverse effects on plant growth, physiology and biochemical properties of garden cress

**DOI:** 10.1038/s41598-021-94860-8

**Published:** 2021-08-02

**Authors:** Ertan Yildirim, Melek Ekinci, Metin Turan, Güleray Ağar, Atilla Dursun, Raziye Kul, Zeynep Alim, Sanem Argin

**Affiliations:** 1 Department of Horticulture, Faculty of Agriculture, Atatürk University, 25240 Erzurum, Turkey; 2 Department of Genetics and Bioengineering, Yeditepe University, 34755 Istanbul, Turkey; 3 Department of Biology, Faculty of Science, Atatürk University, 25240 Erzurum, Turkey; 4 Department of Horticulture and Agronomy, Kyrgyz-Turkish Manas University, Bishkek, Kyrgyz Republic; 5 Department of Agricultural Trade and Management, Yeditepe University, 34755 Istanbul, Turkey

Correction to: *Scientific Reports* 10.1038/s41598-021-86991-9, published online 13 April 2021

In the original version of this Article the Acknowledgments section was incomplete.

“We are very grateful to The Atatürk University, Scientific Research Projects Foundation for generous financial support (Project Number FBA-2019-7219).”

now reads:

“We are very grateful to The Atatürk University, Scientific Research Projects Foundation (Project Number FBA-2019-7219) and Humintech GmbH for generous support.”

The original Article has been corrected.

